# The Complete Mitochondrial Genomes of Five Nivanini Species (Hemiptera: Cicadellidae: Evacanthinae) With Phylogenetic Analysis

**DOI:** 10.1002/ece3.70413

**Published:** 2024-10-11

**Authors:** Wei Wang, Lina Jiang, Ran Li, Sai Jiang, Yongcheng Liu, Jichun Xing, Yujian Li

**Affiliations:** ^1^ School of Life Sciences Qufu Normal University Qufu China; ^2^ Institute of Entomology Guizhou University Guiyang China

**Keywords:** Cicadellidae, codon usage bias, mitochondrial phylogeny omics, structure

## Abstract

To delve deeper into the phylogenetic relationships within Cicadellidae and the taxonomic arrangement of Evacanthinae, our study focuses on the mitochondrial genome sequencing of five Nivanini species: *Extensus latus*, *Concavepiana hamulusa*, *Sophonia nigrilineata*, *Sophonia microstaina,* and *Sophonia fuscomarginata*. The results showed that the length of the five mitochondrial genomes ranged from 15,610 to 16,032 bp and included 37 typical genes. The A + T content of Nivanini ranged from 72.5% to 78.7%, which is consistent with that of other sequenced Evacanthinae species. All transfer RNAs (tRNAs) exhibit the typical cloverleaf secondary structure, except for *trnS* (AGY), which lacks the dihydrouracil (DHU) arm. With regard to protein‐coding genes, all started with ATN codons, except for *atp8*, and most of them use TAA or TAG as termination codons. Using the Bayesian inference and maximum likelihood methods, a phylogenetic tree based on all 37 genes was constructed, with a total of 57 Cicadellidae species and two outgroups included as research objects. The analyses confirmed the monophyletic nature of each subfamily, highlighting Deltocephalinae as the oldest, distinctively parallel to the others.

## Graphical abstract

We conducted a comprehensive analysis by sequencing the mitogenomes of five Nivanini species and integrating this data with previously obtained sequencing results. Our investigation focused on exploring the characteristics of mitogenomes and establishing phylogenetic relationships.

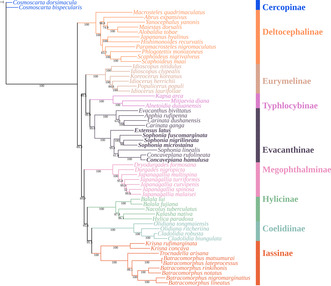

## 1 Introduction

Despite its limitations such as limited data capacity and high heterogeneity compared with nuclear genes, the mitochondrial genome offers certain advantages in specific types of genetic research due to its faster evolutionary rate, maternal inheritance, lack of introns, and most importantly, ease of sequencing (Ballard and Whitlock 2004). Furthermore, the mitogenomes harboring more genome‐level characteristics, such as base content, gene composition, and gene secondary structure, compared to a single gene. As a result, it contains a wealth of genetic information that can be analyzed. These features have found wide applications in the fields of phylogeny, phylogeography, and species identification (Anderson et al. 1981).

Leafhoppers, members of a broader Hemiptera group, use their piercing‐sucking mouthparts to consume plant juices, playing a significant role among agricultural pests. In recent years, an increasing number of phylogenetic studies have been conducted on insects using mitogenomes (Ma et al. 2022; S. Jiang et al. 2022; Y. Jiang et al. 2022; Wang et al. 2022). Despite their great diversity, the mitogenomes of leafhoppers is currently poorly understood. At present, most studies on Evacanthinae focus on morphology. Dietrich (2004) conducted a systematic phylogenetic study using morphological data, confirming the monophyly of Evacanthinae and establishing a higher‐level classification system comprising four tribes. In a study by Wang et al. (2017), a combination of morphological data and molecular biology was employed to elucidate both internal and external phylogenetic relationships within Evacanthinae at the genus level. This study is fairly comprehensive and effectively addresses most relationships within the subfamily; however, it is hindered by issues such as inadequate species representation and low support rates for certain nodes, particularly those pertaining to the genus *Sophonia* (Wang, Dietrich, and Zhang 2017). A comprehensive phylogenomic investigation (encompassing 388 nucleotide sequences) examined the monophyletic and polyphyletic characteristics of various subfamilies within the leafhopper family. However, it faced challenges related to low resolution, which hindered the clarification of certain deep internal branches within this phylogenetic grouping (Dietrich et al. 2017; Hu et al. 2023). Due to insufficient support and data, the phylogenetic relationship in this group has not been completely resolved. Among the complete or nearly complete mitogenomes data of Cicadellidae included by NCBI, most of them are concentrated in Deltocephalinae and Typhlocybinae. Evacanthinae comprises a total of eight species, with six species belonging to the Evacanthini tribe and two species to the Nivanini tribe (data up to September 2023). In contrast, these tribes collectively account for 30 genera and 266 described species for Evacanthini, and 16 genera with 88 described species for Nivanini. The lack of adequate mitogenomes data has limited understanding of the molecular evolution and genetic diversity of this subfamily. Therefore, sequencing the mitogenomes of Evacanthinae species will help enrich phylogenetic studies of Cicadellidae (Hemiptera).

In this study, we used high‐throughput sequencing technology to investigate five complete mitogenomes of Nivanini: *Extensus latus*, *Concavepiana hamulusa*, *Sophonia nigrilineata*, *Sophonia microstaina*, *Sophonia fuscomarginata*, including the only *Extensus* created by Huang (1989). Using the available data and additional complete mitogenomes sequences obtained from other Evacanthinae species in GenBank, our study aimed to elucidate the characteristics and structure of mitogenomes in the five Evacanthinae species, while also investigating the phylogenetic relationships among them. Furthermore, the inclusion of these newly sequenced complete mitogenomes contributes to the enrichment of the genome databases for both Cicadellidae and Evacanthinae, pointing to their potential utility as a valuable resource for future investigations into phylogenetic relationships.

## 2 Materials and Methods

### 2.1 Sample Collection and DNA Extraction

Adult specimens of five species were obtained from two provinces in China in 2022 (Table 1). All samples were collected from the field and did not require any permits. The collected specimens were preserved in 100% ethanol and stored in a laboratory refrigerator at −80°C in the School of Life Sciences, Qufu Normal University. The identification relied on their morphological characteristics (Li, Li, and Xing 2020; Chen, Yang, and Li 2012). The SanPrep DNA GelExtraction kit (Sangon, Shanghai, China) was utilized to extract DNA from the tissues of three specimens. To eliminate interference from the intestinal microbiota during sequencing, the abdominal regions of all samples were removed for subsequent treatment. The male genital specimens and DNA samples are currently archived at the College of Life Sciences, Qufu Normal University, Shandong Province, China.

**TABLE 1:** Sampling details for five specimens used in this study.

Species	Location	Collection date
*Extensus latus*	Wenshan Zhuang and Miao Autonomous Prefecture, Yunnan Province (23°21′ N, 103°54′ E)	July 30, 2022
*Sophonia nigrilineata*	Wenshan Zhuang and Miao Autonomous Prefecture, Yunnan Province (23°21′ N, 103°54′ E)	July 31, 2022
*Sophonia microstaina*	Wenshan Zhuang and Miao Autonomous Prefecture, Yunnan Province (23°21′ N, 103°54′ E)	July 27, 2022
*Concavepiana hamulusa*	Guilin City, Guangxi Zhuang Autonomous Region (25°47′ N, 110°1′ E)	July 15, 2022
*Sophonia fuscomarginata*	Wenshan Zhuang and Miao Autonomous Prefecture, Yunnan Province (23°21′ N, 103°54′ E)	July 25, 2022

### 2.2 Sequence Analysis

Genomic DNA from all samples was sent to Personalbio Inc. (Shanghai, China) for library construction and next generation sequencing (NGS). A library with an average insertion fragment size of 400 bp was constructed using the Illumina TruSeq DNA sample preparation kit (Illumina, San Diego, CA, USA). The Illumina NovaSeq platform was then used for full‐run (2 × 150 PE) 150 bp pair‐end sequencing of all constructed libraries. After filtering, about 2G of clean data with high quality were used for subsequent genome assembly. We use the MITOS BLAST search on the web server and the NCBI use invertebrates genetic code comments assembly sequence (Altschul et al. 1997), MITOZ v.1.04 web server (Meng et al. 2019) was used to identify the locations of 22 typical tRNAs and predict their secondary structure. All rRNA genes were identified based on the location of neighboring tRNA genes and comparison with mitogenomes sequences of other leafhopper preserved at NCBI. In addition, the relative synonymous codon Usage (RSCU) value and number of codons were calculated using MEGA v11.0.13 (Tamura, Stecher, and Kumar 2021). The amino acid content heat map is plotted using TBtools v2.019 (Chen et al. 2020). Finally, the asymmetry of the chain is calculated using the following formula: AT skew = (A − T)/(A + T) & GC skew = (G − C)/(G + C) (Perna and Kocher 1995). Nucleotide diversity (Pi) values were determined using sliding window analysis (a sliding window of 200 bp and a step size of 25 bp) in DnaSP V6.12 (Rozas et al. 2017). The average rate of nonsynonymous substitutions (Ka), the average rate of synonymous substitutions (Ks), and the average ratio of Ka/Ks of each PCG were calculated with DnaSP v6.12.

### 2.3 Phylogenetic Analysis

Fifty‐nine representative species from eight subfamilies, including five newly sequenced species, *Extensus latus* (OQ957162.1), *Concavepiana hamulusa* (OR727344.1), S*ophonia nigrilineata* (OR727342.1), *Sophonia microstaina* (OR727343.1), and *Sophonia fuscomarginata* (OR727345.1), were selected and phylogenetic trees were reconstructed (Table 2). The concatenated nucleotide sequences of the complete set of genes, including 13 PCGs, 22 tRNA genes, and 2 rRNA genes, were utilized for phylogenetic analysis. Two species of Cercopoidea, *Cosmoscarta bispecularis* (KP064511.1), and *Cosmoscarta dorsimacula* (NC040115.1), were designated as outgroups. For each PCGs and RNA sequence alignment, MAFFT v7.250 (Katoh and Standley 2013) was used for the iterative refinement method L‐INS‐I, the resulting alignments are evaluated and manually corrected using the MEGA v11.0.13 program, and connected via PhyloSuite v1.2.3 (Zhang et al. 2020). The best‐fit substitution models (GTR + F + I + G4) of nucleotide sequences were tested by ModelFinder (Kalyaanamoorthy et al. 2017). For maximum likelihood (ML) analysis, a phylogenetic tree was constructed by IQ‐TREE v1.6.12 (Nguyen et al. 2015) with the standard bootstrap approximation approach, involving 5000 replicates. BI analysis was performed using MrBayes v3.2.6 (Ronquist et al. 2012) with four chains. Two independent runs of 2,000,000 generations were carried out with sampling every 1000 generations. The first 25% of trees were discarded as burn‐in. After the average standard deviation of split frequencies fell below 0.01 and the potential scale reduction factor (PSRF) approached 1.0, stationarity was assumed. The phylogenetic trees that arose from the analysis were displayed and enhanced using iTOLv6.7.4 (Letunic and Bork 2021).

**TABLE 2:** Sequences used in this study.

Subfamily	ID	Organism
Cercopinae (family)	KP064511.1	*Cosmoscarta bispecularis*
	NC040115.1	*Cosmoscarta dorsimacula*
Coelidiinae	MN780581.1	*Olidiana ritcheriina*
	NC057966.1	*Olidiana tongmaiensis*
	NC067788.1	*Cladolidia biungulata*
	NC067789.1	*Cladolidia robusta*
Deltocephalinae	KY039116.1	*Alobaldia tobae*
	KY364883.1	*Hishimonoides recurvatis*
	KY817243.1	*Scaphoideus maai*
	KY817244.1	*Scaphoideus nigrivalveus*
	MT332315.1	*Phlogotettix monozoneus*
	NC036131.1	*Yanocephalus yanonis*
	NC036296.1	*Maiestas dorsalis*
	NC036298.1	*Japananus hyalinus*
	NC039560.1	*Macrosteles quadrimaculatus*
	NC045238.1	*Abrus expansivus*
	NC045270.1	*Paramacrosteles nigromaculatus*
Eurymelinae	MN935487.1	*Idiocerus herrichii*
	MZ169558.1	*Koreocerus koreanus*
	NC029203.1	*Idioscopus nitidulus*
	NC039427.1	*Populicerus populi*
	NC039642.1	*Idioscopus clypealis*
	NC039741.1	*Idiocerus laurifoliae*
Evacanthinae	KX437723.1	*Sophonia linealis*
	MK948205.1	*Evacanthus acuminatus*
	OR727342.1	*Sophonia nigrilineata*
	OR727343.1	*Sophonia microstaina*
	OR727344.1	*Concavepiana hamulusa*
	OR727345.1	*Sophonia fuscomarginata*
	MN227168.1	*Concaveplana rufolineata*
	NC077601.1	*Carinata dushanensis*
	NC077602.1	*Apphia rufipenna*
	NC077613.1	*Evacanthus bivittatus*
	NC082036.1	*Carinata ganga*
	OQ957162.1	*Extensus latus*
Hylicinae	MG813488.1	*Balala lui*
	NC056920.1	*Hylica paradoxa*
	NC056921.1	*Balala fujiana*
	NC056922.1	*Kalasha nativa*
	NC056923.1	*Nacolus tuberculatus*
Iassinae	NC036480.1	*Trocnadella arisana*
	NC045858.1	*Batracomorphus lateprocessus*
	NC046067.1	*Krisna concava*
	NC046068.1	*Krisna rufimarginata*
	NC080324.1	*Batracomorphus lineatus*
	NC080325.1	*Batracomorphus matsumurai*
	NC080326.1	*Batracomorphus nigromarginattus*
	NC080327.1	*Batracomorphus notatus*
	NC080328.1	*Batracomorphus rinkihonis*
Megophthalminae	NC035684.1	*Durgades nigropicta*
	NC035685.1	*Japanagallia spinosa*
	NC072571.1	*Dryodurgades formosana*
	NC082332.1	*Japanagallia curvipenis*
	NC082333.1	*Japanagallia multispina*
	NC082334.1	*Japanagallia turriformis*
	NC082340.1	*Japanagallia malaisei*
Typhlocybinae	NC069628.1	*Alnetoidia dujuanensis*
	NC069862.1	*Kapsa arca*
	OK448489.1	*Mitjaevia diana*

## 3 Results

### 3.1 Mitogenomes Structure and Organization

**FIGURE 1:**
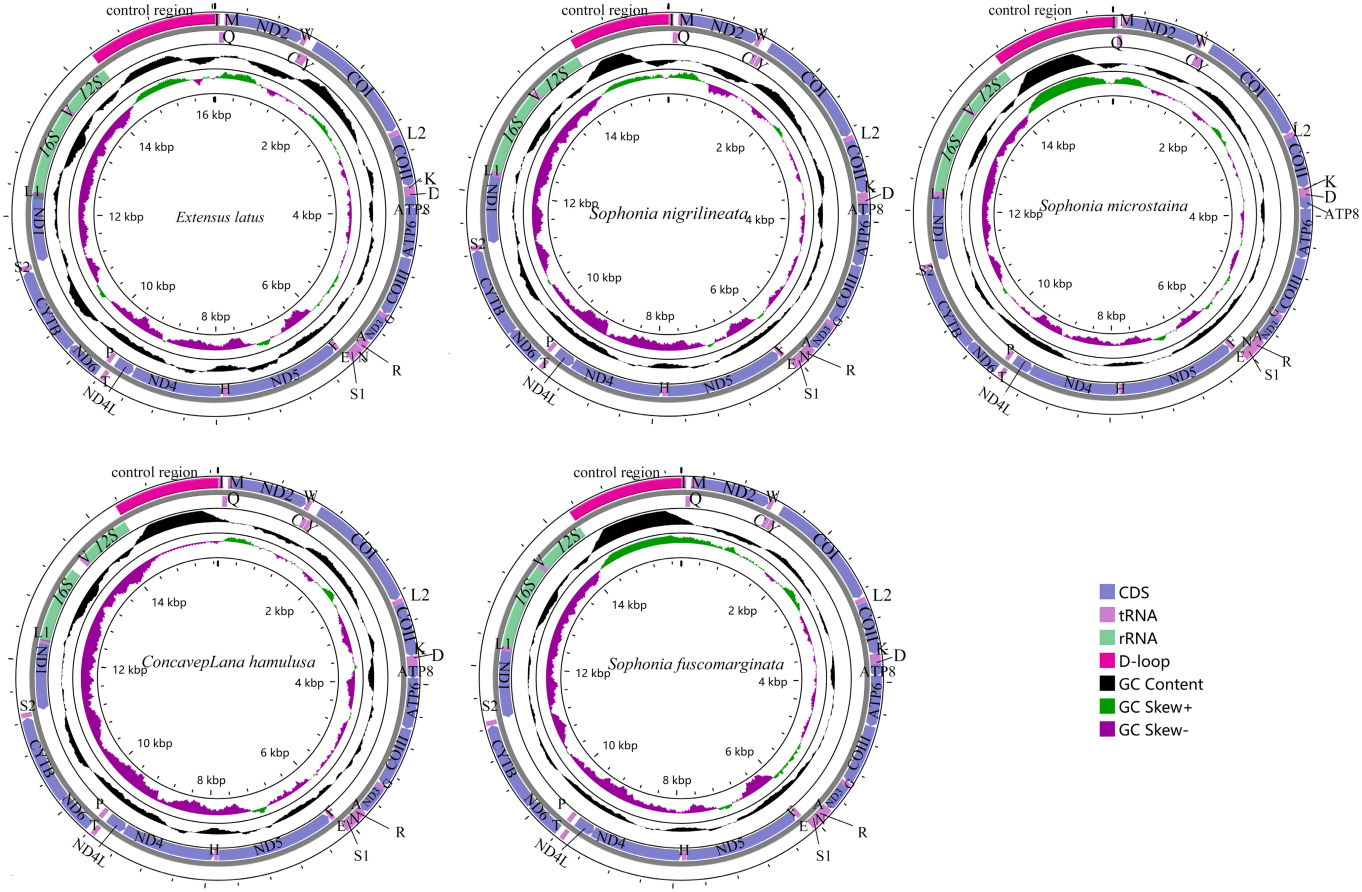
Mitogenomes of *Extensus latus*, *Concavepiana hamulusa*, *Sophonia nigrilineata*, *Sophonia microstaina*, and *Sophonia fuscomarginata*.

**FIGURE 2:**
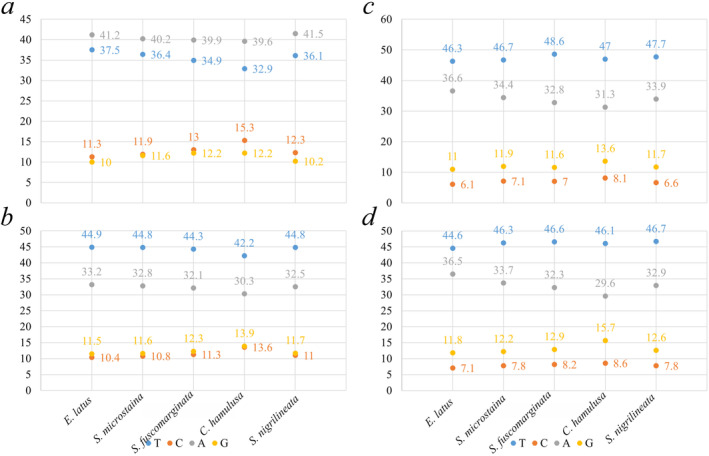
Basic composition of the mitogenomes of five species. (a) All, (b) PCGs, (c) RnaS, (d) RnaL.

The mitogenomes of the five species analyzed in this study were all circular molecules, ranging in length from 15,610 bp (*S nigrilineata*) to 16,032 bp (*E latus*). Detailed annotations and circular maps were found in Figure 1 and Table 3. These mitogenomes consisted of 37 genes, including 13 PCGs, 22 transfer RNA genes (tRNAs), and two ribosomal RNA genes (rRNAs). Additionally, similar to other sequenced Cicadellidae species, they also possess a control region with high A + T content (Luo et al. 2019; Zhou et al. 2016; Wu et al. 2016). Among these 37 genes, 23 were located on the heavy chain (H chain) while 14 were found on the light chain (L chain). The base compositions of the mitogenomes of the five species analyzed showed a predominance of A and T nucleotides (Figure 2a). Specifically, the mean base composition values were 40.48% A, 12.76% C, 11.24% G, and 35.56% T, respectively. The combined content of A and T (A + T) ranged from 72.5% to 78.7%, which was significantly higher than the G + C content. This high A + T content was a characteristic feature of mitogenomes in Hemiptera species.

**TABLE 3:** Organization of the five species mitochondrial genomes.

Gene	Position		Size	Intergenic nucleotides	Codon		Strand
	From	To			Start	Stop	
*Extensus latus/Concavepiana hamulusa/Sophonia fuscomarginata/Sophonia microstaina/Sophonia nigrilineata*							
trnI	1/1/1/1/1	62/65/63/63/63	62/65/63/63/63	0/0/0/0/0			H/H/H/H/H
trnQ	60/63/61/61/61	127/130/128/128/128	68/68/68/68/68	−3/−3/−3/−3/−3			L/L/L/L/L
trnM	127/130/128/128/128	194/196/193/192/193	68/67/66/65/66	−1/−1/−1/−1/−1			H/H/H/H/H
nad2	195/197/194/193/194	1166/1168/1162/1161/1162	972/972/969/969/969	0/0/0/0/0	ATA/ATA/ATA/ATA/ATA	TAA/TAG/TAG/TAG/TAA	H/H/H/H/H
trnW	1169/1171/1161/1160/1161	1232/1231/1221/1222/1222	64/61/61/63/62	2/2/−2/−2/−2			H/H/H/H/H
trnC	1225/1224/1214/1215/1215	1287/1284/1278/1275/1274	63/61/65/61/60	−8/−8/−8/−8/−8			L/L/L/L/L
trnY	1289/1292/1286/1276/1284	1351/1354/1347/1337/1345	63/63/62/62/62	1/7/7/0/9			L/L/L/L/L
cox1	1356/1357/1350/1345/1351	2891/2892/2885/2880/2886	1536/1536/1536/1536/1536	4/2/2/7/5	ATG/ATG/ATG/ATG/ATG	TAA/TAA/TAA/TAA/TAA	H/H/H/H/H
trnL2	2898/2895/2895/2883/2889	2961/2959/2959/2947/2953	64/65/65/65/65	6/2/9/2/2			H/H/H/H/H
cox2	2965/2960/2963/2948/2954	3640/3638/3638/3626/3632	676/679/676/679/679	3/0/3/0/0	ATA/ATT/ATT/ATT/ATT	T/T/T/T/T	H/H/H/H/H
trnK	3641/3639/3639/3627/3633	3711/3709/3709/3697/3702	71/71/71/71/70	0/0/0/0/0			H/H/H/H/H
trnD	3711/3710/3710/3698/3704	3777/3774/3772/3757/3765	67/65/63/60/62	1/0/0/0/1			H/H/H/H/H
atp8	3778/3775/3772/3758/3765	3930/3927/3924/3910/3917	153/153/153/153/153	0/0/−1/0/−1	TTG/TTG/TTG/TTG/TTG	TAA/TAA/TAA/TAA/TAA	H/H/H/H/H
atp6	3924/3921/3918/3904/3911	4580/4577/4574/4560/4567	657/657/657/657/657	−7/−7/−7/−7/−7	ATG/ATG/ATG/ATG/ATG	TAA/TAA/TAA/TAA/TAG	H/H/H/H/H
cox3	4585/4579/4576/4562/4569	5363/5358/5355/5341/5348	779/780/780/780/780	4/1/1/1/1	ATG/ATG/ATG/ATG/ATG	TA/TAA/TAA/TAA/TAA	H/H/H/H/H
trnG	5363/5358/5355/5341/5348	5425/5420/5416/5402/5410	63/63/62/62/63	−1/−1/−1/−1/−1			H/H/H/H/H
nad3	5426/5421/5417/5403/5411	5779/5774/5770/5756/5764	354/354/354/354/354	0/0/0/0/0	ATT/ATA/ATT/ATT/ATA	TAG/TAG/TAG/TAG/TAG	H/H/H/H/H
trnA	5778/5773/5769/5755/5763	5839/5835/5829/5817/5826	62/63/61/63/64	−2/−2/−2/−2/−2			H/H/H/H/H
trnR	5839/5834/5830/5817/5826	5902/5899/5890/5880/5890	64/66/61/64/65	−1/−2/0/−1/−1/0			H/H/H/H/H
trnN	5910/5900/5893/5882/5893	5975/5965/5957/5945/5958	66/66/65/64/66	7/0/2/1/2			H/H/H/H/H
trnS1	5975/5965/5957/5945/5958	6041/6030/6022/6010/6025	67/66/66/66/68	−1/−1/−1/−1/−1			H/H/H/H/H
trnE	6044/6031/6027/6011/6028	6106/6094/6088/6075/6090	63/64/62/65/63	2/0/4/0/2			H/H/H/H/H
trnF	6108/6096/6090/6076/6092	6172/6161/6156/6139/6156	65/66/67/64/65	1/1/1/0/1			L/L/L/L/L
nad5	6173/6163/6157/6140/6157	7835/7828/7822/7805/7822	1663/1666/1666/1666/1666	0/1/0/0/0	ATT/ATT/ATT/ATT/ATC	T/T/T/T/T	L/L/L/L/L
trnH	7840/7835/7829/7812/7829	7904/7898/7892/7873/7892	65/64/64/62/64	4/6/6/6/6			L/L/L/L/L
nad4	7906/7898/7893/7874/7893	9225/9217/9206/9193/9212	1320/1320/1314/1320/1320	1/−1/0/0/0	ATG/ATG/ATG/ATG/ATG	TAG/TAG/TAA/TAA/TAA	L/L/L/L/L
nad4L	9233/9220/9200/9193/9212	9511/9498/9478/9471/9490	279/279/279/279/279	7/2/−7/−1/−1	ATG/ATG/ATG/ATG/ATG	TAA/TAG/TAA/TAA/TAA	H/H/H/H/H
trnT	9514/9501/9481/9474/9493	9576/9566/9543/9538/9561	63/66/63/65/69	2/2/2/2/2			H/H/H/H/H
trnP	9577/9567/9544/9539/9562	9643/9633/9611/9602/9626	67/67/68/64/65	0/0/0/0/0			L/L/L/L/L
nad6	9646/9636/9614/9605/9629	10130/10127/10105/10096/10120	485/492/492/492/492	2/2/2/2/2	ATT/ATT/ATA/ATA/ATT	TA/TAA/TAA/TAA/TAA	H/H/H/H/H
cytb	10130/10120/10098/10089/10113	11266/11256/11234/11225/11249	1137/1137/1137/1137/1137	−1/−8/−8/−8/−8	ATG/ATG/ATG/ATG/ATG	TAA/TAG/TAA/TAG/TAG	H/H/H/H/H
trnS2	11278/11262/11237/11224/11248	11342/11322/11298/11284/11308	65/61/62/61/61	11/5/2/−2/−2			H/H/H/H/H
nad1	11342/11322/11289/11284/11299	12271/12251/12227/12213/12237	930/930/939/930/939	−1/−1/−10/−1/−10	ATT/ATT/ATT/ATT/ATT	TAA/TAA/TAA/TAA/TAA	L/L/L/L/L
trnL1	12272/12295/12294/12280/12299	12337/13364/13506/13483/13486	66/1070/1213/1204/1188	0/−21/1/1/−4			L/L/L/L/L
rrnL	12337/12252/12228/12214/12238	13551/12315/12292/12278/12302	1215/64/65/65/65	−1/0/0/0/0			L/L/L/L/L
trnV	13551/13535/13507/13485/13494	13615/13601/13571/13550/13561	65/67/65/66/68	−1/17/0/1/7			L/L/L/L/L
rrnS	13615/13605/13572/13552/13564	14359/14348/14307/14286/14304	745/744/736/735/741	−1/3/0/1/2			L/L/L/L/L

### 3.2 Protein‐Coding Gene

**FIGURE 3:**
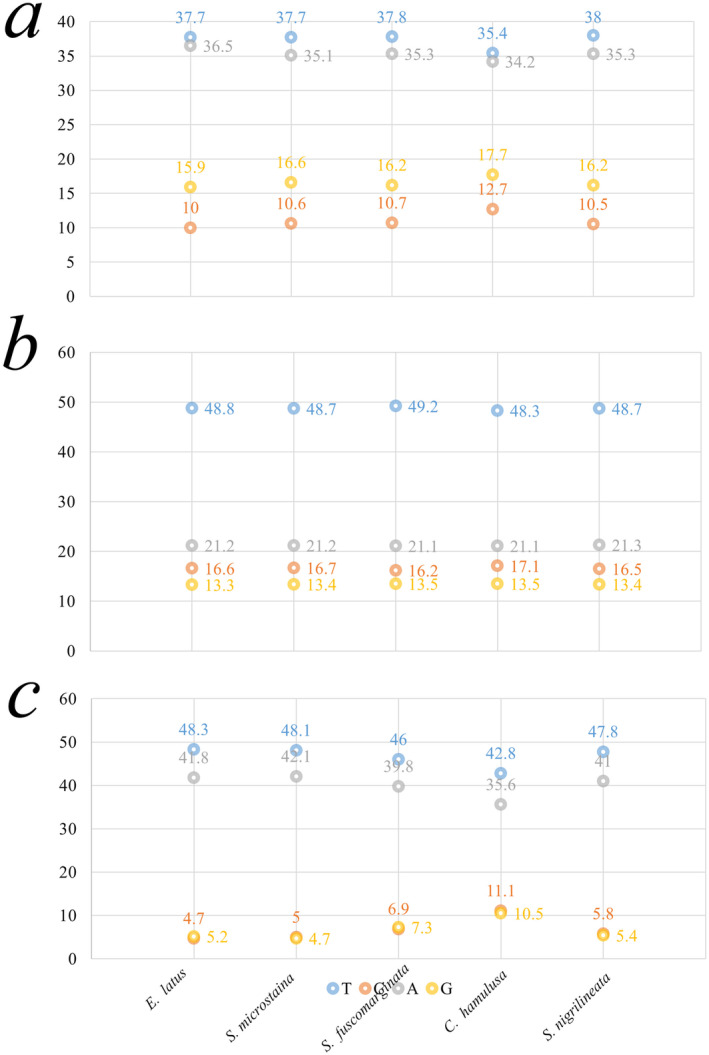
Basic composition of the three positions of the protein‐coding genes (PCGs) in five species. (a) The first codons, (b) the second codons, (c) the third codons.

The base composition, after removing stop codons, showed a higher proportion of T in PCGs, as shown in Figure 2b. Across the entire mitogenomes, A had the highest base composition. In terms of different codon positions, all three positions had a higher content of A and T. Specifically, Positions 1 and 3 had similar proportions of A and T, while Position 2 had a higher proportion of T and a lower proportion of A, as depicted in Figure 3.

**FIGURE 4:**
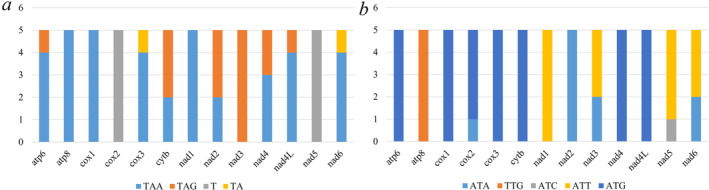
Start and stop codons of the protein‐coding genes (PCGs) in five species. (a) Start codons, (b) stop codons.

The PCGs in all five mitogenomes are initiated by the ATN codon, except for *atp8* (Figure 4a), which is initiated by TTG, as previously reported (Hassan et al. 2023). In terms of stop codons, except for two PCGs (*cox2*, *nad5*), which use incomplete stop codons T, the rest of the PCG stop codons are mostly TAA or TAG (Figure 4b). It is worth noting that TA stop codons also appeared in two PCGs of E. latus (*cox2*, *nad6*). The explanation for this is that during mRNA maturation, incomplete stop codons are modified to the complete TAA codon (Ojala, Montoya, and Attardi 1981) through posttranscriptional polyadenylation.

**FIGURE 5:**
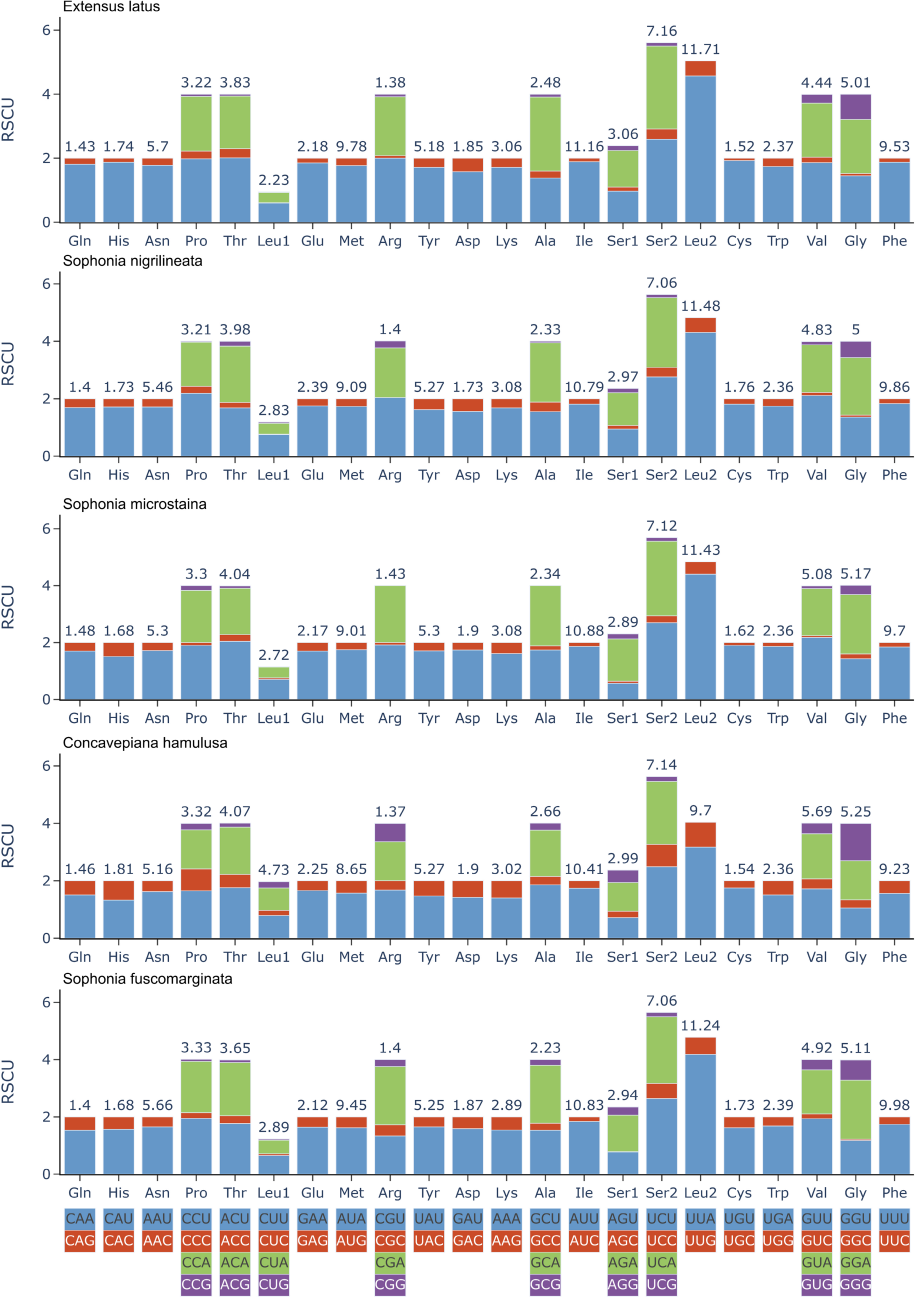
Relative synonymous codon usage values (RSCU) of PCGs in five species.

**FIGURE 6:**
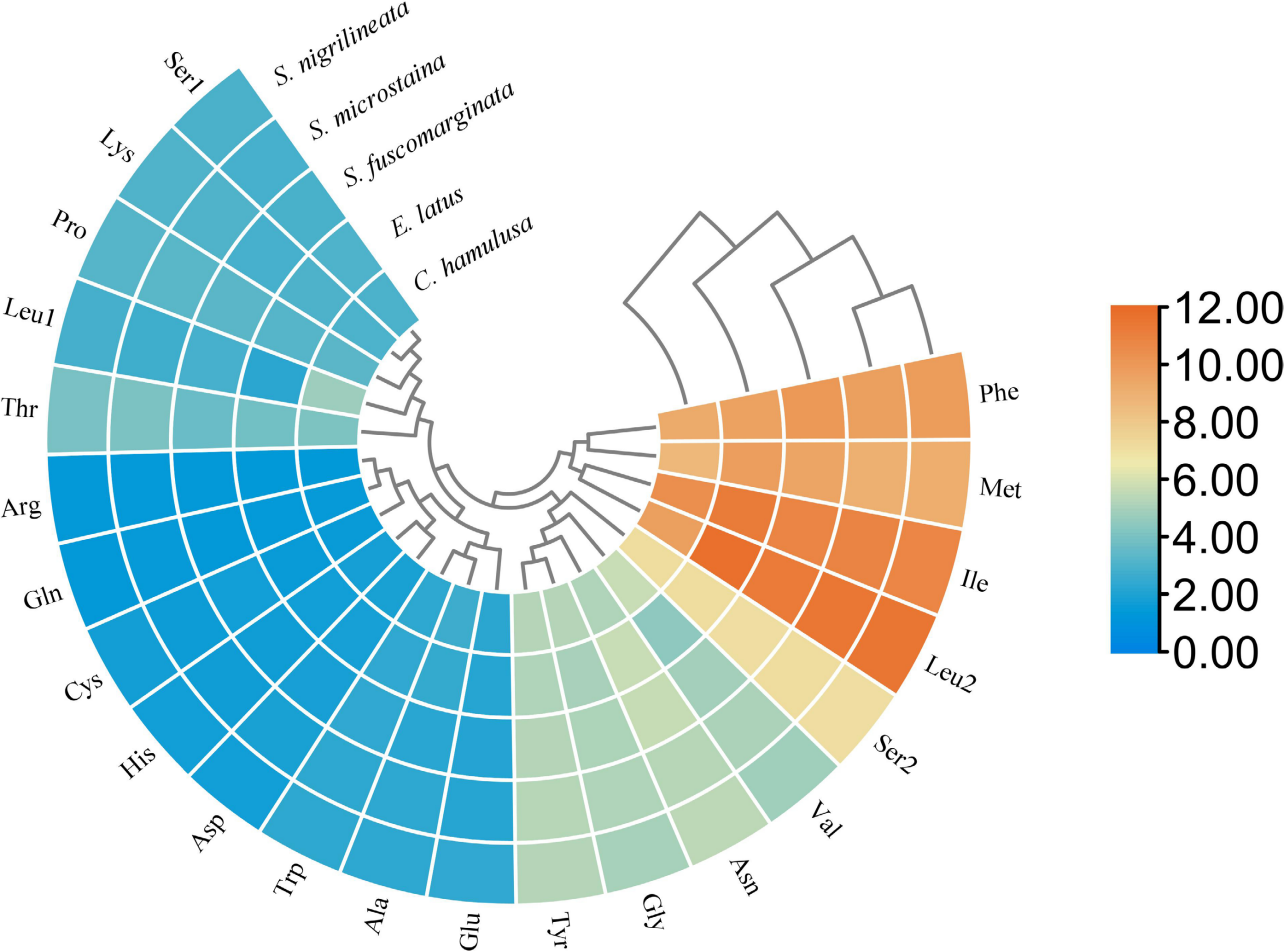
Amino acid composition of the 13 PCGs of five mitogenomes.

Figure 5 depicts the relative synonymous codon use (RSCU) values for the five PCGs found in the mitogenomes of the species under study. The analysis revealed that the RSCU values and codon frequencies were similar across all five species' mitogenomes. Notably, the usage of adenine (A) and thymine (T) was more prevalent compared to guanine (G) and cytosine (C). The four codons with the highest occurrence frequency were AUU (Ile), UUA (Leu), UUU (Phe), and AUA (Met), which collectively accounted for 37.99%–42.26% of the total amino acids (Figure 6).

### 3.3 RNA Gene

Among five mitogenomes examined, the regions containing the rrnS and rrnL genes of rRNA were found to be situated between *trnF* and *trnL* (UUR), with *trnV* serving as the separator (Figure 1). The total length of rrnS ranges from 735 bp (*S. microstaina*) to 745 bp (*E. latus*), and rrnL ranges from 1070 bp (*C. hamulusa*) to 1315 bp (*E. latus*). The A + T content of rrnS ranged from 75.7% (*C. hamulusa*) to 81.1% (*E. latus*), and the variation trend of base composition was T>A>G>C (Figure 2c). The variation trend of base composition in rrnL was T>A>G>C (Figure 2). The content of A + T ranged from 78.3% (*C. hamulusa*) to 82.9% (*E. latus*).

**FIGURE 7:**
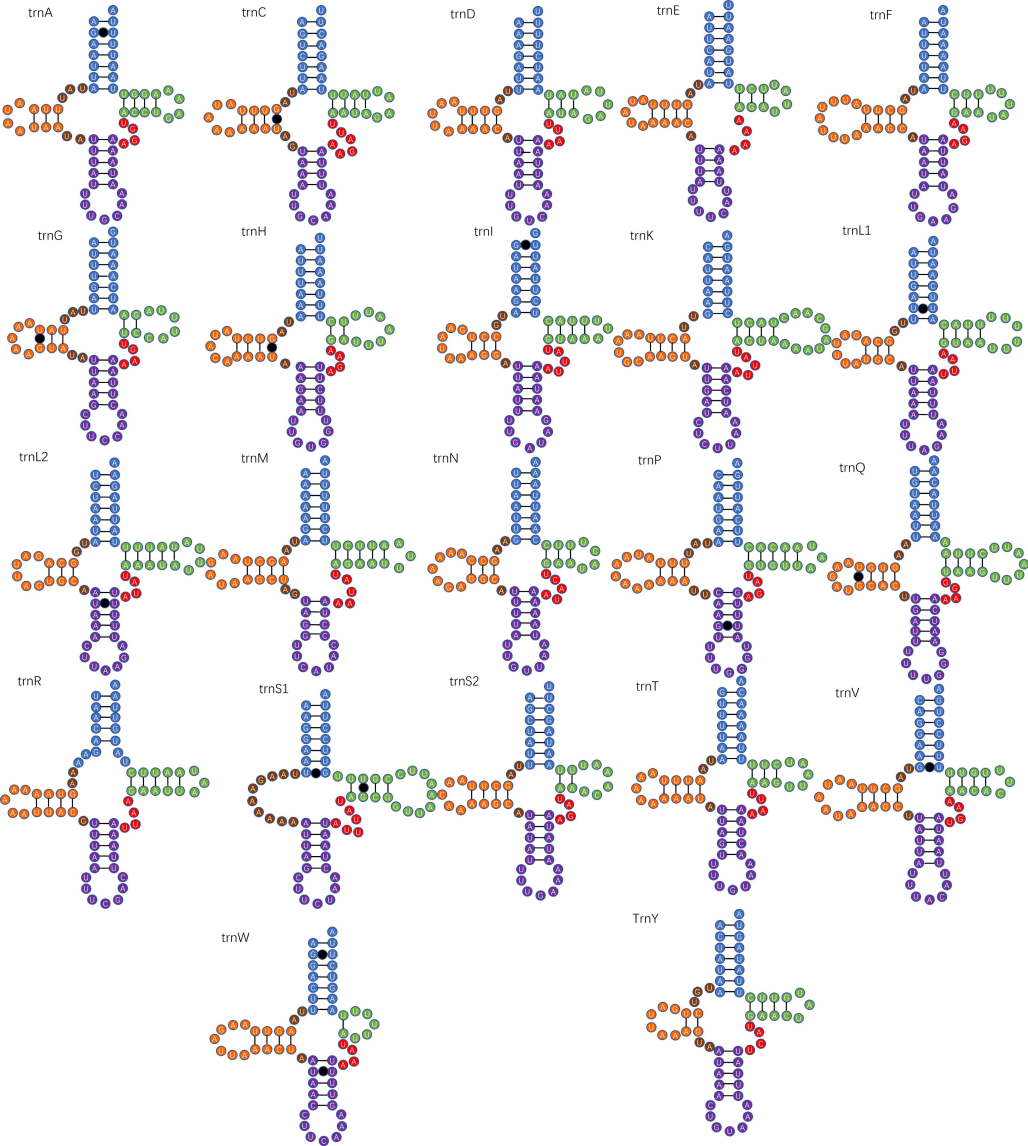
Predicted secondary structures of the 22 tRNAs of *Sophonia fuscomarginata* mitogenome.

Figure 7 Illustrates that all five species' mitochondrial genomes harbor a combined total of 22 transfer RNA (tRNA) genes (The other four species are shown in Figures [Link], [Link]). Out of these, 21 tRNA folded into the classic clover leaf secondary structure. However, *trnS* (AGY) in all species lacked the dihydrouridine (DHU) arm, which is a common observation in most metazoans (Garey and Wolstenholme 1989). Mismatches were found in several tRNA rings, with the G–U type being the most abundant. Other types of mismatches, such as U–U in *trnL1* and C–U in *trnC*, were also present. These mismatches can be corrected through the RNA editing process (Lavrov, Brown, and Boore 2000).

When comparing tRNA structures across the five species, it was evident that the stem sequence showed a higher level of conservation compared to the loop sequence. Among the four branches of tRNA, the DHU arm exhibited the highest conservation, while the AC arm displayed the lowest level. Notably, certain tRNA stems, such as *trnL2* and *trnI*, showed complete sequence conservation, indicating a high level of conservation.

### 3.4 Overlapping and Intergenic Spacers

All gene gaps and overlaps are listed in Table 3. 13 (*C. hamulusa*, *S. fuscomarginata*, *S. microstaina*)‐16 (*S. nigrilineata, E. latus*) intervals were found in the five species studied in this study, with the length ranging from 1 to 17 bp. 11 (*C. hamulusa*)‐13 (*S. microstaina*, *E. latus*, *S. fuscomarginata*, and *S. nigrilineata*) genes were found to overlap, with a length of 1–21 bp. In all five species, it was observed that there are seven identical‐length overlapping regions and one spacer. Furthermore, there are four continuous areas in each of the five species that exhibit no interruptions or overlaps.

### 3.5 Control Region

**FIGURE 8:**
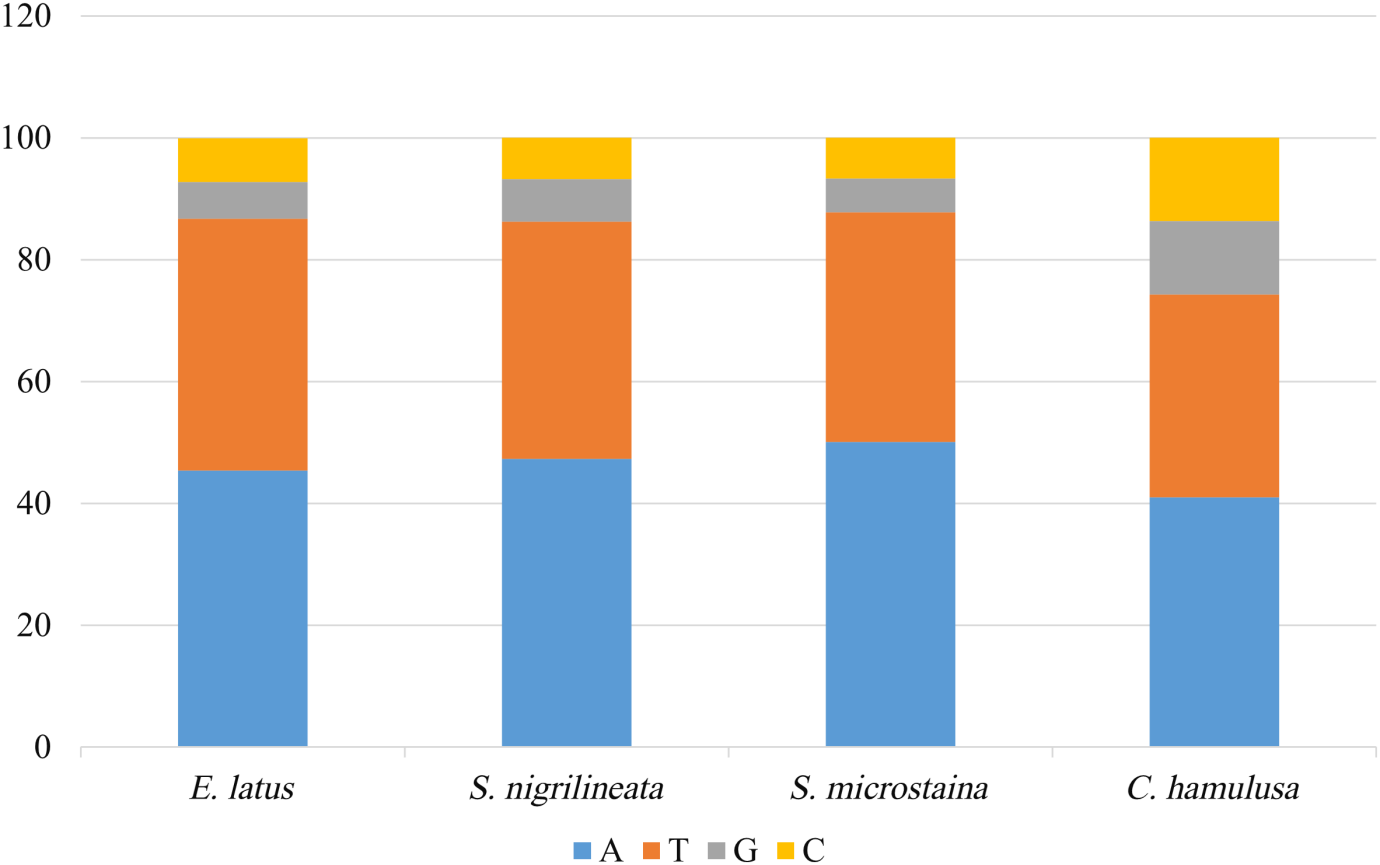
Base composition in the control region (CR) of four species.

**FIGURE 9:**
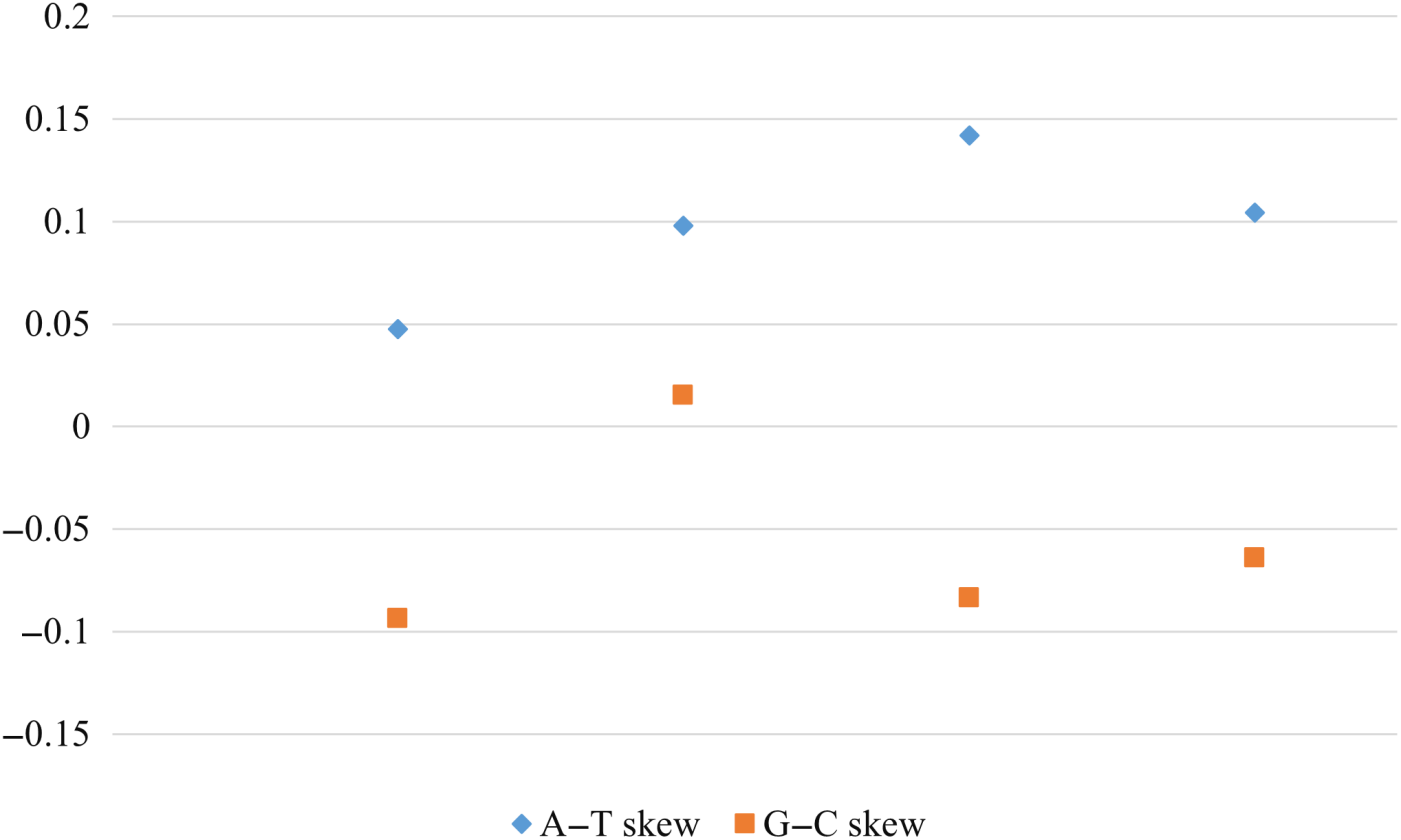
A‐T skew in the control region (CR) of four species.

The CR regions of the five newly sequenced mitogenomes was located between s‐rRNA and *trnI*, and its size ranged from 393 bp (*S. microstaina*) to 916 bp (*C. hamulusa*). It is noteworthy that the position in the *S. fuscomarginat* control region is a sequence formed by the perfect repetition of the same sequence multiple times, calculating its AT content as close to 50%. Such a phenomenon leads us to suspect that this sequence does not originate from the *S. fuscomarginat* control region, hence *S. fuscomarginat* will not be included in the following analysis of the control region. The CR was the longest noncoding region, and the AT content was the highest, ranging from 74.35% (*C. hamulusa*) to 87.79% (*S. microstaina*) (Figure 8). All AT skews were positive (0.048–0.142), while all GC skews were negative (−0.093–0.064) except for *S. nigrilineata* (Figure 9).

### 3.6 Evolutionary Rate

**FIGURE 10:**
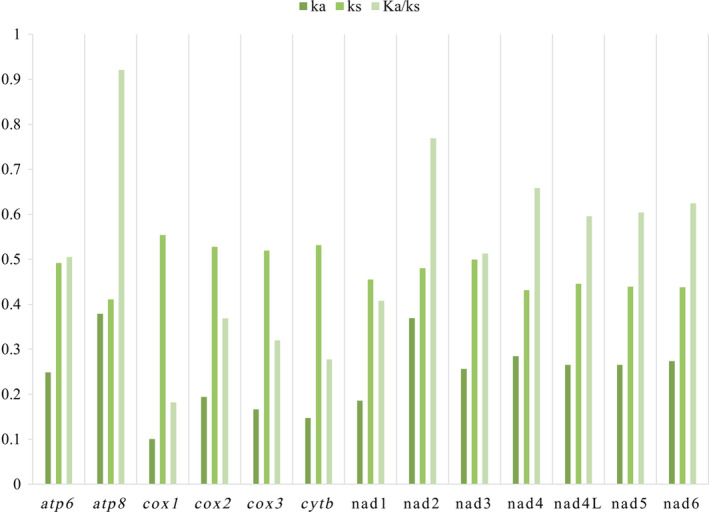
Ratio of nonsynonymous (Ka) to synonymous (Ks) substitution rates of each 13 PCGs among five species.

To measure the selection pressure, the ratio of nonsynonymous substitution rate to synonymous substitution rate (Ka/Ks) was calculated for the 13 PCGs in the mitogenomes of the five species (Figure 10). The results showed that the Ka/Ks values of all 13 PCGs were < 1, ranging from 0.182 for the smallest *cox1* to 0.921 for the largest *atp8*. This indicates that all 13 PCGs were under purification selection.

Interestingly, the *atp8* gene had the largest Ka/Ks value, suggesting that it faces the least selection pressure and evolves faster than the other PCGs in the mitogenomes.

**FIGURE 11:**
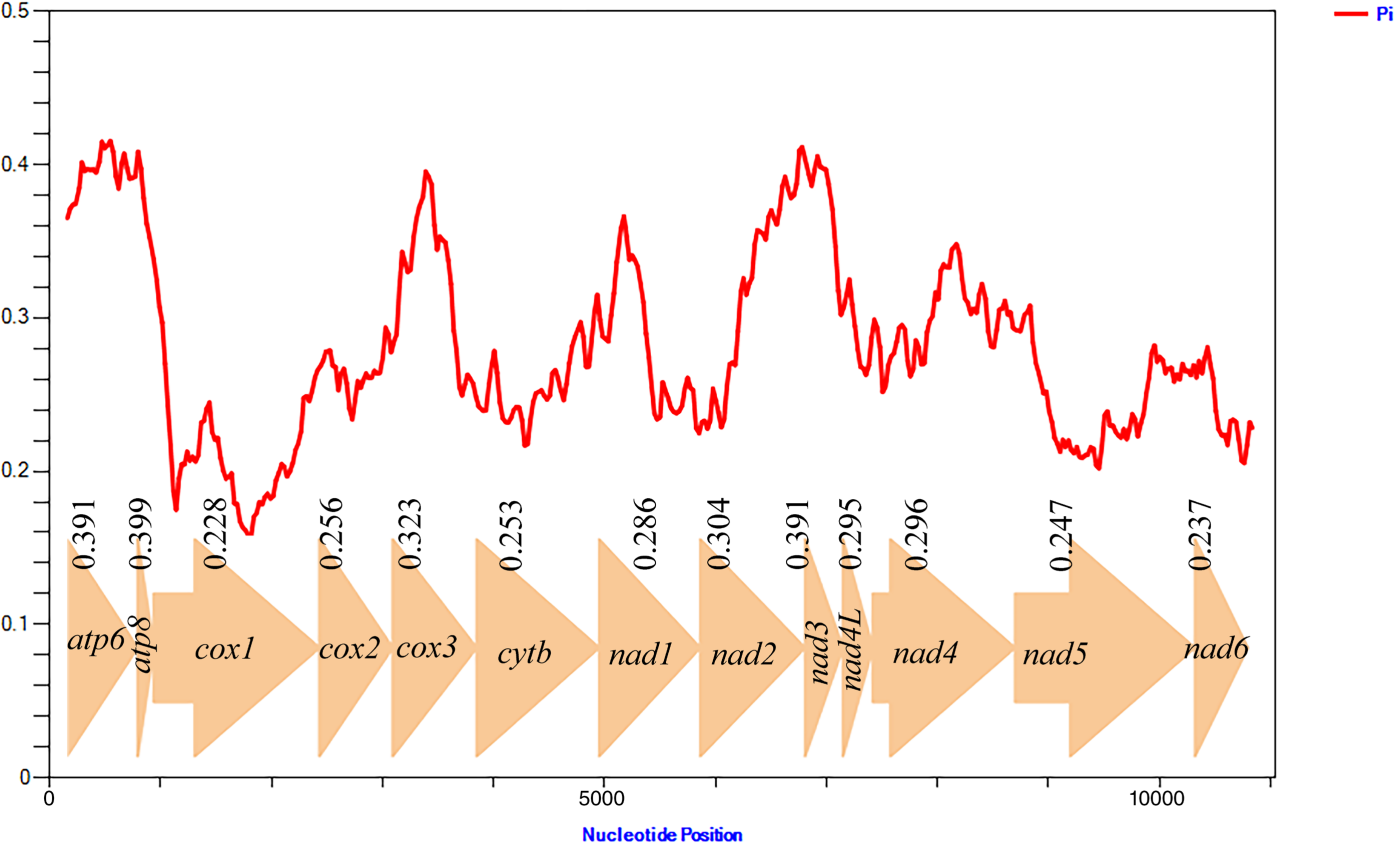
Sliding window analyses based on 13 PCGs of five new sequenced mitogenomes. The red line represents the value of nucleotide diversity (Pi) (200 bp window with 25 bp step).

In Figure 11, the analysis of nucleotide diversity across the 13 PCGs in the five sequenced species revealed a range of values. The *cox1* gene exhibited a nucleotide diversity of 0.228, while the *atp8* gene displayed a higher nucleotide diversity of 0.399.

Comparing the nucleotide diversity among the 13 PCGs, it was found that *atp8*, *nad3*, and *atp6* showed high levels of nucleotide diversity, indicating significant variability. On the other hand, the *cox1* and *nad5* genes were observed to have relatively conservative levels of nucleotide diversity.

### 3.7 Molecular Phylogenetic Analysis

The phylogenetic relationships among 59 species of Cicadellidae were analyzed using whole mitogenomes sequences. Both Bayesian inference (BI) and maximum likelihood (ML) methods were used to reconstruct phylogenetic trees, which showed the same stable topology with high node support values.

**FIGURE 12:**
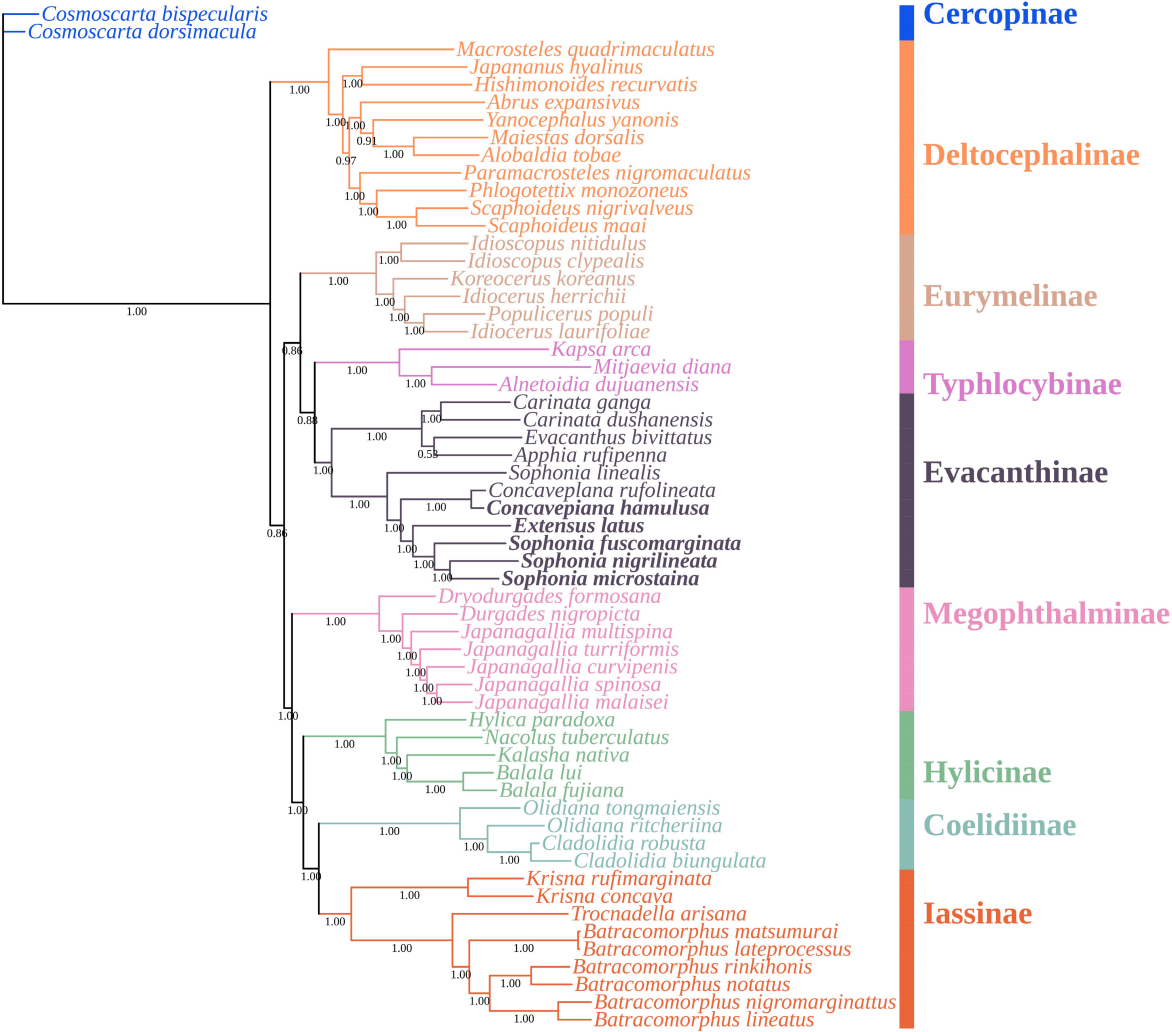
Phylogenetic tree of Cicadellidae species inferred via Bayesian analyses of the whole mitogenomes sequences.

**FIGURE 13:**
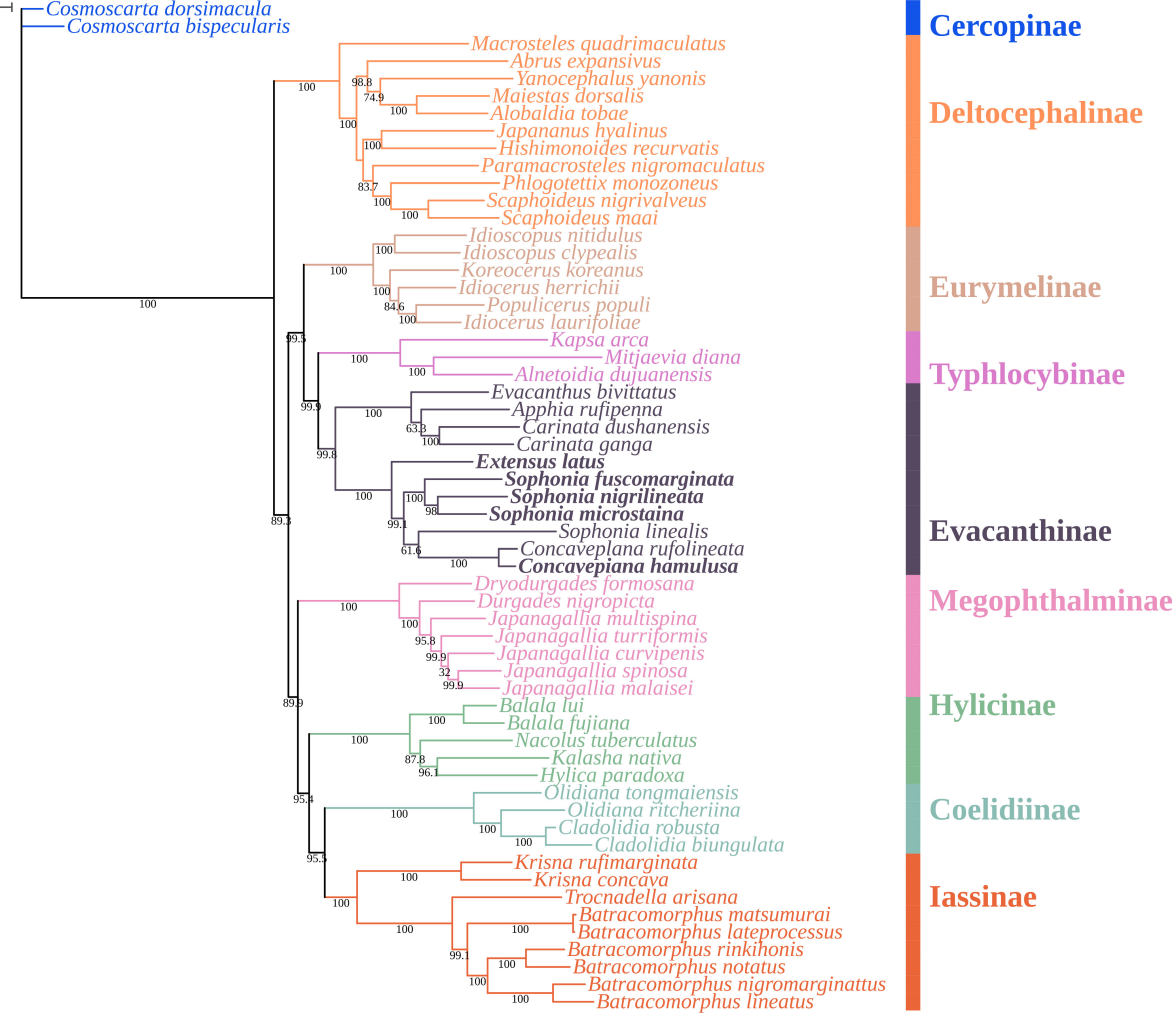
Phylogenetic tree Cicadellidae species inferred via maximum likelihood analyses of the whole mitogenomes sequences.

In Figures 12 and 13, it was observed that the monophyly of each subfamily received strong support in both ML and BI analyses, as follows: ((((Iassinae + Coelidiinae) + Hylicinae) + Megophthalminae) + ((Evacanthinae + Typhlocybinae) + Eurymelinae)) + Deltocephalinae. The relationships among the eight subfamilies were nearly identical in ML and BI analyses, with differences primarily observed within the subfamilies. Typhlocybinae was identified as the sister group to Evacanthinae, with a high node support value (BS = 99.9, PP = 0.88), forming clade I with Eurymelinae and clade II with Megophthalminae, Hylicinae, Cicadellinae, and Iassinae, while Deltocephalinae was placed at the base of the entire tree, consistent with previous studies (Yu and Zhang 2021).

Within Evacanthinae, two parallel clades received high node support (BS = 99.8, PP = 1.00), one comprising *Concavepiana*, *Sophonia*, and *Extensus* (Nivanini), and the other comprising *Evacanthus*, *Apphia*, and *Carinata* (Evacanthini), supporting the monophyly of Evacanthini and Nivanini (Wang, Dietrich, and Zhang 2017; Jiang et al. 2024). The recently sequenced Sophonia species clustered together in both maximum likelihood (ML) and Bayesian inference (BI) analyses, distinguishing them from previously identified Sophonia species. Based on this result, we cannot fully ascertain the monophyly or polyphyly of the genus *Sophonia*; however, it is at least not chaotic, which differs from previous studies (Wang, Dietrich, and Zhang 2017). The amalgamation of Concavepiana species with the existing taxa provides initial support for the monophyletic status of Concavepiana. In the case of *Extensus*, it formed a clade parallel to *Sophonia* in the BI analysis and parallel to *Concavepiana* in the ML analysis, with high node support values (BS = 99.1, PP = 1.00). Given that only one species of *Extensus* has been described to date, and the entire Evacanthinae subfamily still lacks sufficient genetic data support, we are unable to make definitive conclusions.

## 4 Discussion

In this study, the researchers focused on sequencing and comparing the complete mitogenomes of five species from three genera. Among these species, only one belonged to the genus *Extensus*.

The mitochondrial genome sequences of the five species examined in this study are all circular molecules containing 37 genes and a high A + T region. All five mitochondrial genomes exhibit ATN start codons for the PCGs, except for *atp8*, which initiates with TTG. The majority of PCGs terminate with TAA or TAG codons, with the exception of two genes (*cox2* and *nad5*) which utilize incomplete stop codon “T.” A 7‐base pair overlap was identified between *atp8* and *atp6*, a characteristic observed not only in Evacanthinae species but remains consistent when examining the entire class Insecta (Liu et al. 2018; Luo et al. 2023). Despite both *atp8* and *atp6* displaying relatively high evolutionary rates, the overlap between them appears to be notably stable at least in terms of sequence conservation. The classical cloverleaf secondary structure was identified in all tRNAs, except for *trnS1*, which lacks the DHU arm.

The mitogenomes of these five newly sequenced species were compared with those of previously sequenced Evacanthinae species. The comparison revealed that the overall length of tRNA and PCGs was similar among the species, with the main length variations occurring in rrna (ribosomal RNA) and CR (control region). Interestingly, the *cox1* gene, which is commonly used as a genetic marker (Demari‐Silva et al. 2015), showed the lowest nucleotide diversity (Pi) and Ka/Ks values in this study.

In order to gain a better understanding of their evolutionary relationships, we conducted initial investigations using mitochondrial genome data. Based on concatenated nucleotide sequences of all 37 genes, we reconstructed the phylogenetic relationships within 8 subfamilies of Cicadellidae. Our Bayesian inference (BI) and maximum likelihood (ML) analyses revealed the following topology: ((((Iassinae + Coelidiinae) + Hylicinae) + Megophthalminae) + ((Evacanthinae + Typhlocybinae) + Eurymelinae)) + Deltocephalinae. Although the clade comprising Iassinae, Coelidiinae, Hylicinae, and Megophthalminae is positioned alongside the clade of Evacanthinae, Typhlocybinae, and Eurymelinae (BS = 89.3, PP = 0.86), the absence of corroborative support from prior studies precludes a definitive confirmation of their sister relationship. We have notably confirmed the monophyly of the tribes Evacanthini and Nivanini, consistent with findings from morphology‐based studies (Dietrich 2004). It is important to note that this study only analyzed two out of four tribes, which is insufficient. Therefore, more comprehensive research and increased sampling of mitochondrial genome sequences are warranted.

Although the overall topology of the phylogenetic tree was similar to previous studies (Wang, Wang, and Dai 2021; Tang, Huang, and Zhang 2020), there were slight differences. These discrepancies may arise from variations in the molecular data used, differences in species selection for tree construction, and the limited availability of mitogenomes databases. Nevertheless, this study contributes valuable insights into the relationships between Evacanthinae and other subfamilies, while also expanding the mitogenomes database for the Nivanini subgroup within Evacanthinae. The researchers suggested that future studies should include a wider range of genotypes and taxa to address these issues and improve the understanding of phylogenetic relationships within the Cicadellidae family.

## Author Contributions

**Wei Wang:** formal analysis (equal), writing – original draft (equal). **Lina Jiang:** formal analysis (equal), resources (equal). **Ran Li:** methodology (equal), resources (equal), software (equal). **Sai Jiang:** supervision (equal), validation (equal). **Yongcheng Liu:** validation (equal), visualization (equal). **Jichun Xing:** investigation (equal), methodology (equal), software (equal). **Yujian Li:** funding acquisition (equal), resources (equal), supervision (equal).

## Conflicts of Interest

The authors declare no conflicts of interest.

## Supporting information


Figure S1.

Figure S2.

Figure S3.

Figure S4.

## Data Availability

The data supporting the findings of this study are openly available from the National Center for Biotechnology Information at https://www.ncbi.nlm.nih.gov (accessed on 13 April 2024), accession numbers: OR727342.1–OR727345.1, OQ957162.1.
