# Supplementary figures and images for: The Complete Mitochondrial Genomes of Five Nivanini Species (Hemiptera: Cicadellidae: Evacanthinae) With Phylogenetic Analysis
Source: Ecol Evol. 2024 Oct 11;14(10):e70413. doi: 10.1002/ece3.70413 (PMC11470156; doi:10.1002/ece3.70413)

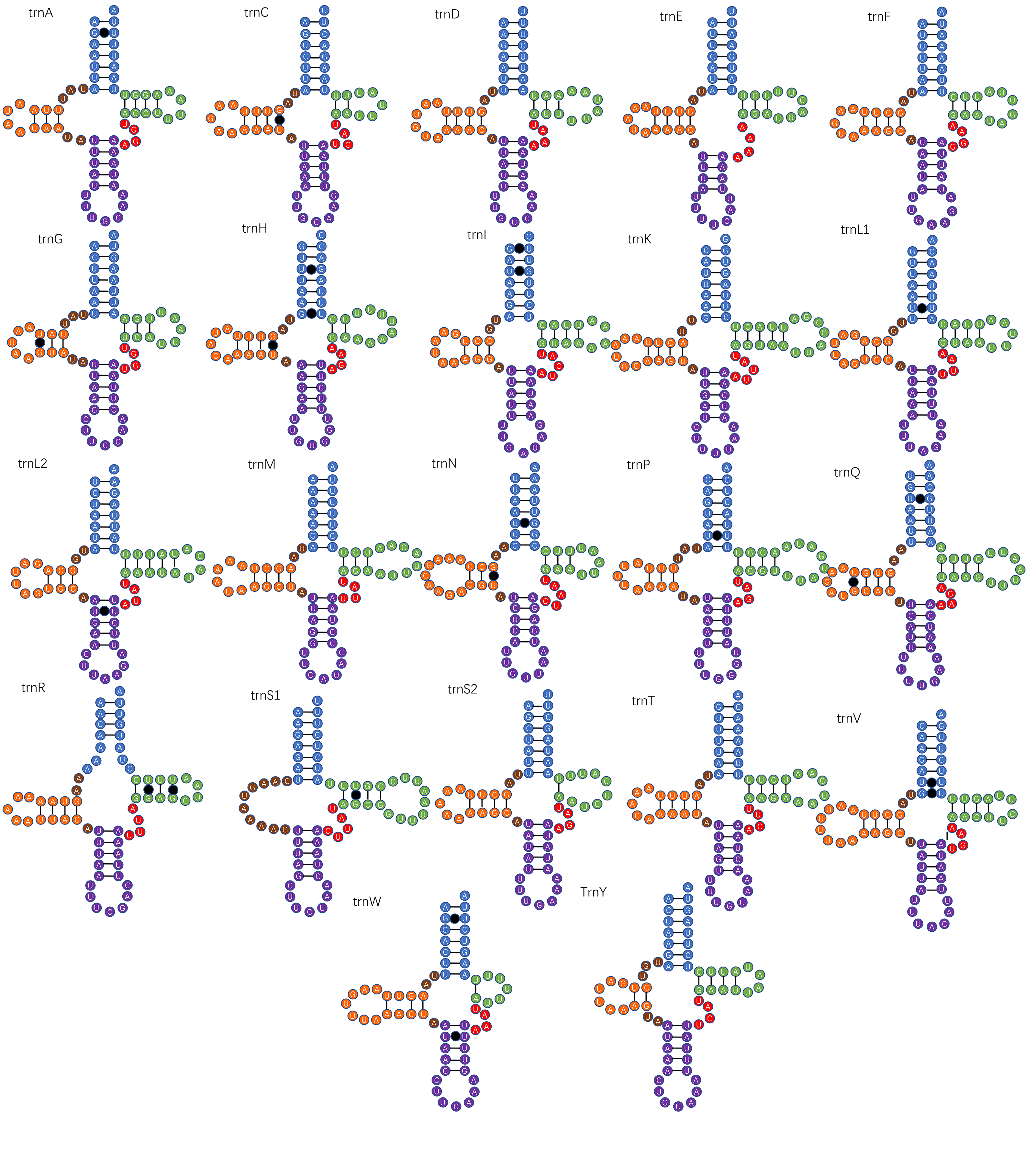

Supplement: Supplementary file 1 — Figure S1. [file ECE3-14-e70413-s003.jpg]

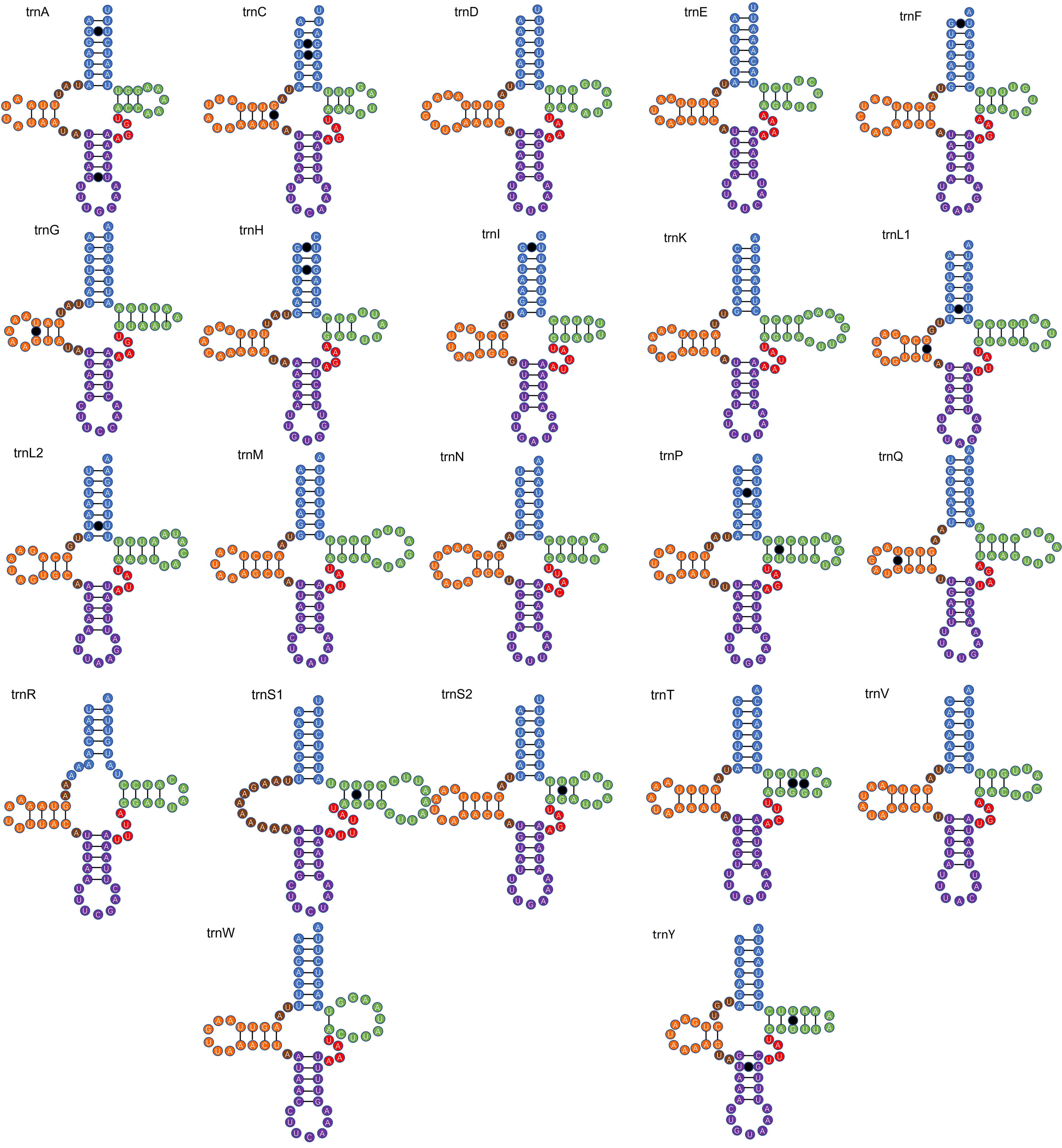

Supplement: Supplementary file 2 — Figure S2. [file ECE3-14-e70413-s004.jpg]

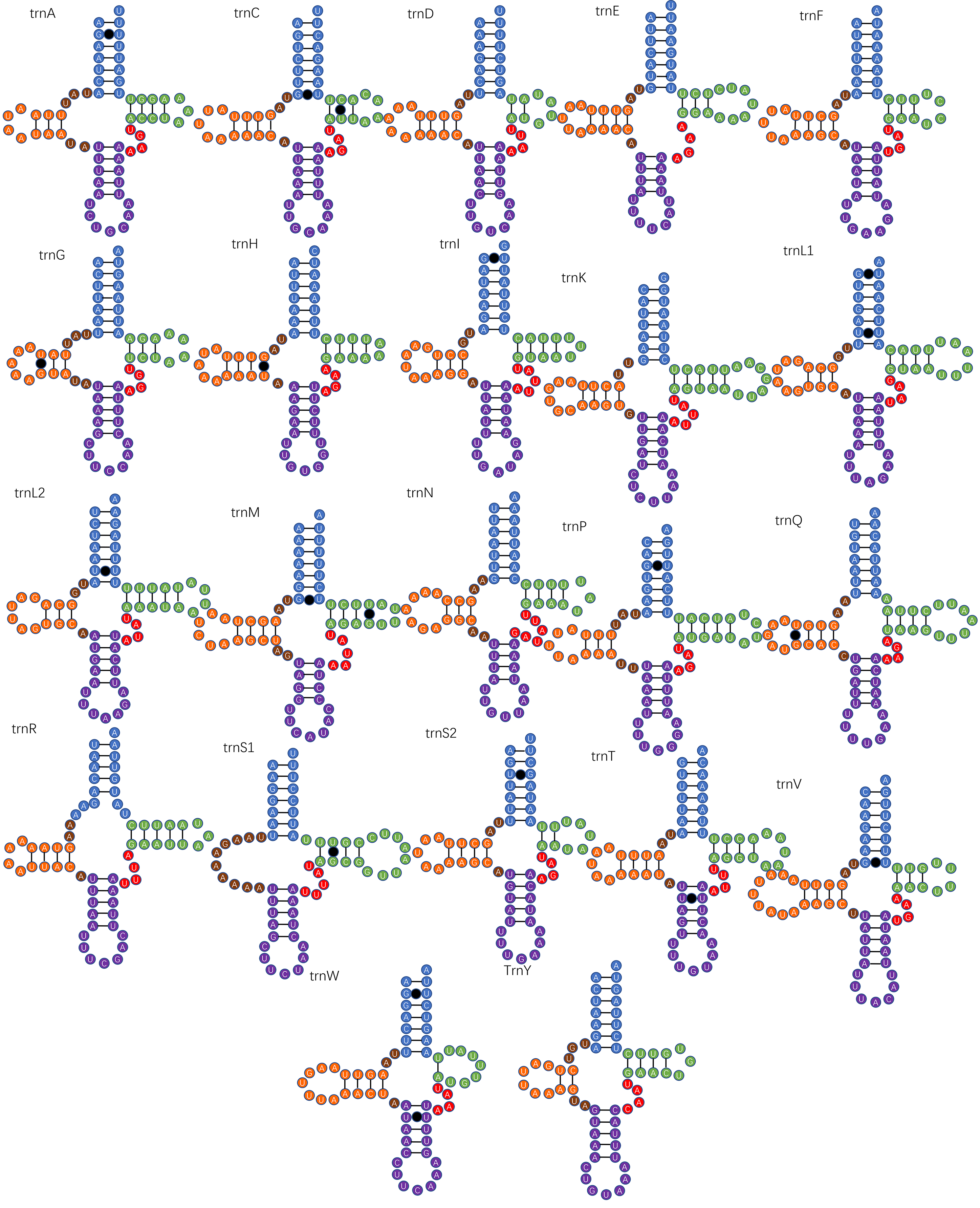

Supplement: Supplementary file 3 — Figure S3. [file ECE3-14-e70413-s001.jpg]

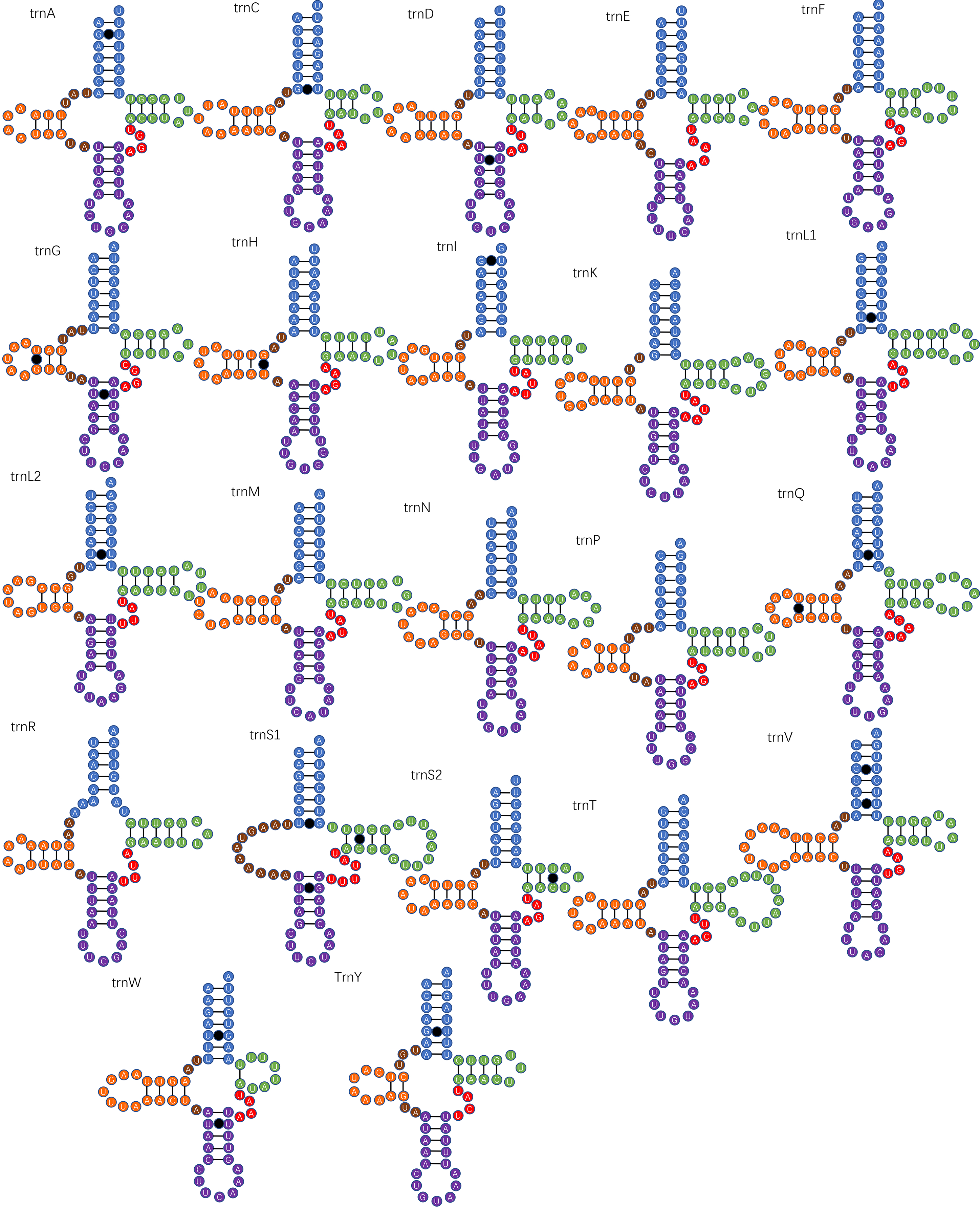

Supplement: Supplementary file 4 — Figure S4. [file ECE3-14-e70413-s002.jpg]
